# Artificial Intelligence in Surgical Risk Prediction

**DOI:** 10.3390/jcm12124016

**Published:** 2023-06-13

**Authors:** Stamatios Kokkinakis, Evangelos I. Kritsotakis, Konstantinos Lasithiotakis

**Affiliations:** 1 Department of General Surgery, School of Medicine, University Hospital of Heraklion, University of Crete, 71500 Heraklion, Greece; stamatioskokkinakis@gmail.com; 2 Laboratory of Biostatistics, School of Medicine, University of Crete, 71003 Heraklion, Greece; e.kritsotakis@uoc.gr

Risk prediction and stratification of short-term and long-term postoperative outcomes are growing in importance and scope of application in everyday clinical practice. The surgical community increasingly acknowledges the benefit in utilizing risk prediction models to facilitate comparative audits and communication of risks, shared decision-making and informed consent before surgery. Amongst multiple patient outcomes targeted by surgical prognostic models, postoperative complications and prognosis of survival in neoplastic disease are of utmost importance.

Regression-based methods have long been used for developing accurate and interpretable risk prediction models, many of which have established a place in patient management. The traditional culture of statistical modelling requires defining model predictors using subject matter knowledge and pre-specifying an equation formula (such as the logistic model or the Cox proportional hazards model) that dictates how the predictor variables (patient-related features) affect the predicted risk. However, model- or theory-driven approaches may not always be feasible or desirable, and more flexible machine learning (ML) approaches may be preferred.

The ML approach generally downplays the role of context and prior knowledge by relying heavily on the data alone. A typical ML approach uses a computationally intensive algorithm to “learn” from the data in a non-parametric manner, so that it lets the data find the best fitting formula for linking the predictors with the outcome risk. A special form of ML, artificial intelligence (AI), continues iteratively to learn and train (update) the model when it senses further improvement is possible. The ML/AI algorithmic framework may thus range from supervised learning requiring involvement from the analyst to entirely unsupervised learning that automatically identifies patterns in the data without human input. Unsupervised learning is commonly implemented as neural networks (also known as deep learning), which can be considered as a nonlinear extension of logistic regression. In general, a neural network algorithm uses the predictor variables to construct new variables called “neurons” in a number of “hidden” layers. The first hidden layer is a weighted linear combination of the original predictors and the subsequent hidden layers are linear combinations of the neurons of the previous layers. Non-linearity can be introduced by means of a function such as the logit function. The neural network learns iteratively from datasets; the errors from the initial prediction for the patients are fed back into the algorithm and layer weights are recalculated to minimize the error the next time predictions are made. This approach is more flexible in recognizing patterns in the data compared to a standard logistic regression model, but the iterative learning process needs to stop before it produces overly trained (overfitted) predictions that are tailored too much to the peculiarities of the training data and may not generalize for future patients. To mitigate the risks of overfitting, methods that penalize (shrink) the regression weights are frequently employed, such as ridge regression or least absolute shrinkage and selection (LASSO) regression [1].

Several ML techniques have been employed to predict postoperative mortality and morbidity outcomes, with authors often testing multiple options in efforts to find the one best performing. Recently, Cho et al. used data from two large and diverse cohorts of patients undergoing general surgeries to develop ML models with various techniques, including deep neural networks, extreme gradient boosting (XGB), LASSO logistic regression and random forest (RF) algorithm [2]. Biomarkers available immediately after surgery were used as predictors in an effort to identify derangements from the normal postoperative course on time. RF was superior in predicting 30-day mortality, with the area under the receiver operating characteristic curve (AUROC) equal to 0.82 in the validation dataset. Apart from discriminatory ability, the calibration was also examined by the Hosmer–Lemeshow test and by plotting calibration curves [2]. Large databases of non-cardiac surgery patients were utilized to derive ML models for 30-day mortality, using XGB, RF, LASSO regression and naïve Bayes approaches [3]. The XGB model presented high AUROC values of 0.96 and 0.93 in internal and external validation, respectively, and outperformed a traditional logistic regression model. Calibration curves together with a value of the Integrated Calibration Index of 0.0017 in external validation indicated a very small weighted average difference between observed and predicted probabilities [3].

AI-based approaches were recently applied to predict survival probabilities for multiple types of malignancies. A convolutional neural network (CNN) was used in Japan to derive a prognostic model for recurrence following hepatectomy for solitary hepatocellular carcinoma [4]. More than 500 patients were enrolled and CT images were used as a predictor along with several clinical variables. The model had moderate discrimination, with the CT image being the most discriminative predictor for differentiating between high- and low-risk patients [4]. Liu et al. sought to find combinations of clinical variables with treatment modalities that may lead to improved survival in patients with advanced liver cell cancer [5]. The resultant decision tree used three preoperative variables, the alpha fetoprotein, the glutamic oxaloacetic transaminase and the total bilirubin, to classify patients in different survival groups. The technique also provided insight into which treatment options may result in better survival in each group [5]. Similarly, an ML-based model identified patients who would benefit from upfront surgery or neoadjuvant chemotherapy followed by surgery for liver metastases in terms of survival [6]. A RF algorithm was used to identify the best possible treatment for each patient, and classification and regression trees (CARTs) were employed to identify key preoperative factors affecting allocation to surgery or chemotherapy [6]. CARTs were also used in a cohort of patients with intrahepatic cholangiocarcinoma undergoing hepatectomy to classify them in risk groups for recurrence-free survival and overall survival [7]. Simple biomarkers, including CRP and CA 19-9, were used to stratify the patients into three groups and significant differences were noted between the groups in Kaplan–Meier curves, for recurrence-free and overall survival. Using similar curves plotted with the use of the AJCC staging system, a lower Akaike Information Criterion (AIC), implying a better overall model fit and a higher c-index, were noted with the classification built by machine learning [7]. Lin et al. used a random survival forest (RSF), a variant of the RF algorithm designed to handle right-censored survival times, to derive predictions of cancer-specific survival for postoperative pancreatic cancer patients after applying the LASSO method to select predictor variables [8]. The RSF algorithm performed favorably compared to a Cox proportional hazards model and a neural network in terms of discrimination and calibration abilities, as well as in decision curve analysis. That work enables future external validation studies by making the source code of the RSF algorithm openly available [8].

The number of publications involving AI-based prognostic models will likely increase in the coming years, due to increased computing power and storage, advances in AI-assisted research and interest in incorporating these tools into every aspect of modern healthcare. Surgeons wishing to use AI-based tools for risk predictions in their practice should be aware of both their strengths and limitations. ML algorithms have appeared with surprisingly satisfactory predictive performances in many studies, but head-to-head comparisons against traditional regression models have generally shown no significant predictive advantage, if proper methods are applied in both approaches. Moreover, ML methods are not immune to small sample sizes and actually may require truly “big data” to ensure stable predictions [9]. Inadequate sample sizes may have led to overly optimistic performance measures in many studies, as the issue of overfitting may not have been properly addressed. Another key issue with ML-based prediction models is transparency and interpretability by clinicians. The benefit from ML-based approaches is in their ability to analyze large quantities of high-dimensional, diverse and unstructured data that would be extremely difficult to analyze with conventional statistics. However, this advantage can come at the expense of complexity, leading to black box models that are difficult to present in forms understandable by the practicing surgeon. This is in contrast with regression-based models and decision trees, which are inherently interpretable approaches. The lack of transparency and interpretability in AI-based models may additionally hamper the conduct of external validation studies to assess their generalizability in diverse patient populations. A framework for structured quality assessment of AI-based prediction models is still missing, and new research in this domain is needed for improving the quality of published research and ensuring the safe and responsible application of surgical risk prediction models in healthcare [10].
